# Predictors of the Intention to Be Vaccinated against COVID-19 in a Sample of Italian Respondents at the Start of the Immunization Campaign

**DOI:** 10.3390/jpm12010111

**Published:** 2022-01-14

**Authors:** Alessandro Santirocchi, Pietro Spataro, Marco Costanzi, Fabrizio Doricchi, Clelia Rossi-Arnaud, Vincenzo Cestari

**Affiliations:** 1 Department of Psychology, Sapienza University, 00185 Rome, Italy; alessandro.santirocchi@uniroma1.it (A.S.); fabrizio.doricchi@uniroma1.it (F.D.); clelia.rossi-arnaud@uniroma1.it (C.R.-A.); 2 Department of Economy, Universitas Mercatorum, 00100 Rome, Italy; pietro.spataro@unimercatorum.it; 3 Department of Human Sciences, Lumsa University, 00193 Rome, Italy; m.costanzi@lumsa.it

**Keywords:** COVID-19 vaccine, vaccine acceptance, vaccine intention

## Abstract

COVID-19 vaccines are the most promising means of limiting the pandemic. The present study aims at determining the roles of several psychological variables in predicting vaccination intention in Italy. An online questionnaire was disseminated between 9 March and 9 May 2021. The sample included 971 participants. Results showed that most of the participants were willing to vaccinate. Acceptance rates were correlated with age, marital status, and area of residence. Intention to be vaccinated was positively correlated with perceived risk, pro-sociality, fear of COVID-19, use of preventive behaviors, and trust in government, in science, and in medical professionals. Intention to be vaccinated was negatively associated with belief in misinformation. The degree of acceptance is likely to be a result of the campaign tailored to address people’s negative attitudes towards vaccines. Trust in government and trust in science were among the strongest psychological predictors of vaccination intention. Fear of COVID-19, but not perceived risk, was associated with increased vaccine uptake, suggesting that the affective component of risk perception was more important than the cognitive component in predicting participants’ behaviors. Belief in misinformation was associated with reduced vaccination intention. Future studies will take into consideration these variables, to better understand the multifaceted process underlying vaccination intention.

## 1. Introduction

To date, different vaccines against COVID-19 have been approved by regulatory agencies and are currently in use. Worldwide differences among countries exist in the type of vaccine approved and administered. Further, in some countries, the type of vaccine administered varies according to the age range of the people inoculated. As new vaccines were commercialized, the intention to get vaccinated rose in many countries. For example, a survey conducted in April 2021 on more than 10,000 respondents [1] showed that the percentage of people who declared to accept the COVID-19 vaccine was very high in Brazil (93%), Mexico (88%), Spain (83%), and China (81%), fairly high in Italy (79%), Canada (78%), Japan (73%), South Korea (72%), and Germany (71%), moderate in Australia (66%), South Africa (62%), and France (58%), and low in the United States (46%) and Russia (41%). Nevertheless, a meta-analysis of 28 nationally representative samples from 13 countries concluded that, as the pandemic progressed, the percentage of people intending to vaccinate decreased (being about 60%) and the percentage of people intending to refuse vaccination increased (being about 20%) [2]. At the moment in which we are writing (15 December 2021), about 8.59 billion doses have been administered globally, but only 56.5% of the world population has received at least one dose of a COVID-19 vaccine [3]. Shares of people vaccinated are high in countries such as United Arab Emirates (99%), Cuba (90%), Portugal (89%), and China (84%), moderate in countries such as France (77%), United Kingdom (75%), Germany, and United States (both 72%), low in countries such as Russia (48%), South Africa (31%), and Egypt (28%), and extremely low in countries such as Kenya (10%), Ethiopia (7%), and Nigeria (4%). In Italy, more than 104 million doses have been administrated, and 85% of the population (47 million people) has been fully immunized with two doses.

Overall, these data suggest that understanding the factors that determine and promote the intention to be vaccinated represents an enduring mission for psychological research. The present study sought to contribute to this field by investigating the intention to get vaccinated in Italy in the period between March and May 2021—that is, shortly after the beginning of the vaccination campaign. There were three primary aims. First, we sought to provide up-to-date information about vaccine acceptance rates (i.e., the percentage of people who are willing to be vaccinated against COVID-19) in Italy. In a previous study by Palamenghi et al. [4] conducted during the early days of the Italian reopening after the first lockdown (May 2020), a sample of 1004 Italian citizens were asked to report their willingness to be vaccinated against COVID-19 “if a vaccine was found” on a scale ranging from 1 (not likely at all) to 5 (absolutely likely). The results showed that about 59% of the respondents were “likely” or “absolutely likely” to vaccinate. Given the recent emphasis in enhancing public trust in COVID-19 vaccination, we expected this estimate to be substantially higher at the beginning of 2021, see [5,6]. In this respect, we must note that the policy adopted by the Italian government to address vaccine hesitancy has been one of the most fruitful, at least in Europe. Generally speaking, Italy has a long-standing tradition of high coverage of vaccinations. However, in the last decade, the frequency of infant immunization has decreased alarmingly, leading to the introduction of a new law, the “Italian National Immunization Prevention Plan 2017–19” (n. 119/2017), which prescribes mandatory vaccinations against ten diseases for preschool and school-aged children [7]. The implementation of the law contributed to an increased awareness of the importance of vaccination in the Italian population [8]. During the COVID-19 pandemic, this awareness was further boosted by the broad diffusion of science-supporting messages from experts about vaccine safety and effectiveness. Pro-vaccine messages are now common in mass media, including TV, radio, magazines, newspapers, and the Internet. In addition, the Italian government has recently approved two types of green COVID-19 certificates: the Basic Green Pass (proving vaccination, recovery from COVID-19 within the last six months, or a negative result for a molecular or antigenic swab in the last couple of days) and the Super Green Pass (only granted to the vaccinated and those who have recovered from the coronavirus in the last six months). The fact that the Super Green Pass is now compulsory for certain categories (including healthcare workers, school teachers, soldiers, and police officers), as well as for accessing an increasing number of activities and services, has produced an additional boost in vaccination rates.

Our second aim was to determine the impact of individual differences in demographic variables on vaccine acceptance rates. In this respect, common findings are that the intention to vaccinate was higher in males than in females [2,6], and higher in older than in younger people [9,10]. However, in the study by Kerr et al. [6], neither age nor gender were found to be significant predictors of vaccine acceptance in a sample of 700 Italian respondents interviewed between March and October 2020. Lastly, the third aim was to determine the roles of a number of psychological variables in predicting the intention to be vaccinated against COVID-19. Following a theoretical framework originally applied to the study of risk perception [11], psychological predictors were selected in order to assess the cognitive (risk perception, pro-sociality), the emotional (fear of COVID-19), the experiential (direct experience, use of preventative measures, misinformation), and the sociocultural (trust in government, trust in science, trust in medical professionals) aspects of the current pandemic [12].

Previous research has provided evidence in support of the involvement of these variables in the prediction of vaccine hesitancy and/or the intention to be vaccinated [6,9,10,13,14,15], however, to our knowledge, few studies have compared the relative importance of each of them within a single study. For example, trust in government and in medical professionals has been repeatedly demonstrated to play a key role in determining COVID-19 vaccine acceptance. A cross-sectional study in 19 countries showed that willingness to get vaccinated ranged from 88.6% (China) to 55.8% (Russia) and was positively and significantly associated with trust in the government [16] (see [17] for similar results in a Canadian sample). However, as suggested by Kerr et al. [6], current research has not considered the possible overlap between different types of trust (trust in government, trust in science, and trust in medical professionals). Perceived risk (i.e., the subjective likelihood of getting the virus) is another variable which has been often called into question in predicting the adoption of preventative behaviors and the acceptance of COVID-19 vaccines [18,19], in line with the predictions following from the Health Belief Model [20] and the Protection Motivation Theory [21]. However, these studies have typically failed to disentangle the roles of the cognitive and affective components of risk perception and many of them did not evaluate fear or worry of COVID-19 [22]. Lastly, for other predictors such as pro-sociality, available evidence is mixed, with some studies reporting significant associations with vaccination intent [23,24], and other studies reporting no association [25].

The aim of the present study was to provide an updated assessment of vaccine acceptance rates in Italy in the period between March and May 2021 at the launch of the vaccination campaign and further investigate the impact of a broad array of demographical and psychological factors in increasing (or decreasing) participants’ willingness to be vaccinated against COVID-19.

## 2. Materials and Methods

### 2.1. Participants

Table 1 reports the demographic characteristics of our sample.

Overall, we recruited 971 Italian-speaking participants, 411 males and 558 females and 2 participants not reporting their gender. Most of our participants were between 18 and 30 years (*N* = 641), were unmarried (*N* = 681), lived with relatives or partners (*N* = 795), resided in Central Italy (*N* = 713), and many had a university degree (*N* = 380, considering both Bachelor’s and Master’s degrees). At the time the study was conducted, 350 participants lived in a white or yellow area (low risk), 128 lived in an orange area (intermediate risk), and 493 lived in a red area (high risk). Shortly after the 2020 lockdown, the Italian government introduced a classification of regions based on white (minimum risk), yellow, orange, and red (maximum risk) color codes. Each color corresponds to the adoption of a gradually increasing number of preventative measures regulating travel possibilities within a single region and between regions, the opening of businesses, restaurants, and places of sports and culture.

When compared with the general Italian population, participants older than 61 years of age, with a high school diploma (or less), married, and living alone in Northern or Southern Italy were underrepresented in our sample (see Table 1). 

### 2.2. Instruments and Measures

#### 2.2.1. Intention to Be Vaccinated

Intention to be vaccinated was measured with two questions taken from Palamenghi et al. [4]: “Are you willing to be vaccinated against COVID-19?” and “Do you think that your family members should be vaccinated against COVID-19?”. For both questions, participants responded on a five-point Likert scale, ranging from “not at all likely” (1) to “absolutely likely” (5). Scores were summed and therefore could range between 2 and 10. Cronbach’s α was good (α = 0.88).

#### 2.2.2. Perceived Risk

Perceived risk was assessed with three questions taken from Dryhurst et al. [12]: “How likely do you think it is that you will be directly and personally affected by the following in the next 6 months?—Catching the coronavirus/COVID-19”, “How likely do you think it is that your friends and family in the country you are currently living in will be directly affected by the following in the next 6 months?—Catching the coronavirus/COVID-19”, and “How much do you agree or disagree with the following statements?—Getting sick with the coronavirus/COVID-19 can be serious”. For the first two questions, participants responded on a seven-point Likert scale, ranging from “not at all likely” (1) to “very likely” (7). For the third question, participants responded on a five-point Likert scale, ranging from “strongly disagree” (1) to “strongly agree” (5). Scores were summed and could therefore range between 3 and 19. Cronbach’s α was acceptable (α = 0.62).

#### 2.2.3. Pro-Sociality

Pro-sociality was investigated with a single item taken from Dryhurst et al. [12]: “To what extent do you think it’s important to do things for the benefit of others and society even if they have some costs to you personally?”. Participants responded on a five-point Likert scale going from “not at all” (1) to “very much so” (5).

#### 2.2.4. Fear of COVID-19

Feelings of anxiety towards COVID-19 were measured with the Italian version of the Fear of COVID-19 Scale FCV-19S [26,27]. This seven-item scale includes items such as “I am most afraid of Coronavirus-19”, “My hands become clammy when I think about Coronavirus-19”, and “When watching news and stories about Coronavirus-19 on social media, I become nervous or anxious”. Participants responded on a 5-point scale ranging from 1 (strongly disagree) to 5 (strongly agree); thus, total scores ranged from 7 to 35. Cronbach’s α was good (α = 0.86).

#### 2.2.5. Direct Experience

Direct experience with Coronavirus was examined with a single item: “Have you ever had, or thought you might have, the Coronavirus/COVID-19?”. Participants had three response options: “Yes, I had COVID-19”, “I thought I might have COVID-19, but I have been tested as negative”, and “No, I never had COVID-19”. Following Dryhurst et al. [12], this item was dichotomized by considering the first two options as “yes” responses (1) and the last option as a “no” response (0).

#### 2.2.6. Use of Preventive Behaviors

The frequency of use of COVID-19 preventive behaviors during the past three months was assessed with 10 items that were extracted from the COVID-19 preventive methods recommended by the WHO and were previously used by Lee et al. [28]. Examples of the items were: “Washed hands regularly using alcohol-based cleanser or soap and water”, “Avoided social gatherings of more than 4 people”, and “Avoided hand-shaking, hugging, and kissing”. Participants indicated the frequency of use of each behavior on a 5-point scale ranging from 1 (Never) to 5 (Always). Thus, total scores ranged from 10 to 50. Cronbach’s α was excellent (α = 0.92).

#### 2.2.7. Misinformation

COVID-19 misinformation was assessed with 12 items taken from Lee et al. [28] and extracted from COVID-19 misinformation reports by the WHO. Examples of the items were: “Masks can be sterilized and reused after steaming with hot water”, “Drinking tea can prevent COVID-19”, “Taking antibiotics can prevent or treat COVID-19”, “Only the elderly would become infected with the COVID-19”, and “COVID-19 is artificially developed”. Participants first indicated whether they had encountered each statement in the last three months (binary responses: yes/no; total scores ranged from 0 to 12). Then, they answered the following question: “Which of the above information do you believe is correct?”. Responses were provided on a 4-point scale including “none” (1), “some are correct” (2), “most are correct” (3), and “all are correct” (4).

#### 2.2.8. Trust in Government

Trust in government was assessed with a single item taken from Dryhurst et al. [12]: “How much do you trust the country’s politicians to deal effectively with the pandemic?”. Participants responded on a seven-point Likert scale going from “not at all” (1) to “very much” (7).

#### 2.2.9. Trust in Science

Trust in science was assessed with a single item taken from Dryhurst et al. [12]: “How much do you trust each of the following?—Scientists”. Participants responded on a five-point Likert scale going from “cannot be trusted at all” (1) to “can be trusted a lot” (5).

#### 2.2.10. Trust in Medical Professionals

Trust in medical professionals was assessed with a single item taken from Dryhurst et al. [12]: “How much do you trust each of the following?—Medical doctors and nurses”. Participants responded on a five-point Likert scale going from “cannot be trusted at all” (1) to “can be trusted a lot” (5).

### 2.3. Procedure

The questionnaire was prepared using Google Forms and disseminated through different social media (including Facebook, Instagram, Twitter, LinkedIn, Telegram, and WhatsApp), in line with the Italian government’s recommendations on limiting face-to-face interactions. All data were collected between 9 March and 9 May 2021—but note that 829 participants (85% of the whole sample) completed the questionnaire by 31 March. We used a snowball sampling strategy: the links were initially shared with a sample of university students who were encouraged to pass them on to others, with a focus on recruiting the general public. The research was approved by the Ethical Committee of the University Sapienza of Rome (Protocol N.0000476) and all respondents signed an informed consent before participating.

### 2.4. Statistical Analyses

Since our variables resulted from the combination of a different number of questions, they had different ranges and needed to be preliminarily standardized by transforming them into z-scores. Z-scores are measured in terms of standard deviations from the mean and thus inform on how many standard deviations a raw score is away from the mean. Positive scores indicate that the participant’s raw score falls above the mean, whereas negative scores indicate that it falls below the mean.

Statistical analyses were performed in three successive steps. First, we investigated whether participants’ intention to be vaccinated (measured in terms of z-scores) differed as a function of the demographic properties of our sample (gender, age, education, marital status, living condition, region, and type of area). A *t*-test for independent samples was used for gender (because it involved only two categories) while between-subject univariate ANOVAs were used for all other variables: when a significant result was obtained, the main analysis was followed by post-hoc pairwise comparisons (using the Bonferroni adjustment), to determine which pairs of the factor categories were significantly different from each other. Second, Pearson’s correlations were computed between the main variables, to assess which factors were associated with participants’ intention to be vaccinated. Lastly, the correlational analysis was followed by a hierarchical regression analysis to determine which variables predicted participants’ intention to be vaccinated. Demographic factors were entered in the first step as a series of dummy variables, to control for their influence. A dummy variable is a numerical variable used in regression analyses to represent different treatment groups. Specifically, participants were given a value of 0 if they were in the reference group (for example “female” for gender) or a 1 if they were in the other group (“male”). For a variable having *k* levels, *k* – 1 dummy variables were necessary to represent all groups. For example, to represent marital status, which has three different levels (single, married, divorced/widowed), two dummy variables were required. Since we chose the “single” category as the reference level, the first dummy variable was coded 1 if participants belonged to the “married” category and 0 if they belonged to the “single” or “divorced/widowed” categories. The second dummy variable was instead coded 1 if participants belonged to the “divorced/widowed” category and 0 if they belonged to the “single” or “married” categories. Psychological variables (perceived risk, pro-sociality, fear of COVID-19, direct experience, use of preventive behaviors, misinformation, trust in government, trust in science, and trust in medical professionals) were included in the model in the second step, to ascertain whether they explained additional variance, over and above the contribution provided by demographic variables. As usual, the results of the regression analysis are presented in terms of *β* coefficients, which describe the mathematical relationships between each independent variable and the dependent variable. More technically, they represent the mean change in the dependent variable for one unit of change in the predictor variable while holding other predictors constant. Each coefficient was associated with a *t*-test and a *p*-value, which indicated whether the relationship between the predictor and dependent variables was statistically significant. If the *t*-test was not significant (*p* > 0.05), then the predictor had no correlation with the dependent variable, i.e., there was no association between the changes in the independent variable and the shifts in the dependent variable. Otherwise, if the *t*-test was significant (*p* ≤ 0.05), then data favored the hypothesis that there was a non-zero correlation, i.e., that changes in the independent variable were associated with changes in the dependent variable at the population level. For all analyses, the *α* level was set to 0.05.

## 3. Results

Descriptive measures for the variables examined in the present study are reported in Table 2.

In relation to our first aim, acceptance of COVID-19 vaccines was substantially high. In fact, 762 participants (78.5%) responded that they were absolutely likely to be vaccinated against COVID-19, whereas only 35 participants (3.6%) responded that they were not at all likely to get vaccination. Pooling together the first two categories (i.e., “not at all likely” and “very unlikely”), a total of 67 participants (6.9%) were hesitant about their own vaccination. Similarly, when asked about their family members, 750 participants (77.2%) responded that they should absolutely be vaccinated, whereas only 19 participants (2.0%) responded that they should absolutely not be vaccinated. Collapsing the first two categories, a total of 55 participants (5.7%) were hesitant about vaccination for their family members.

With respect to our second aim, we found that participants’ intention to be vaccinated differed as a function of three demographic variables. As reported in Table 3, significant differences were observed for: (i) age (post-hoc comparisons revealed that acceptance rates were lower for participants 41–50 years old than for those who were 18–30 years old, *p* = 0.025, or older than 61 years, *p* = 0.039; all other *p* > 0.32), (ii) marital status (post-hoc comparisons revealed that acceptance rates were lower for participants who were married or divorced/widowed than for those who were single, *p* = 0.003 and *p* = 0.004), and (iii) type of area (post-hoc comparisons revealed that acceptance rates were lower for participants who resided in an orange area than for those who resided in white/yellow or red areas, *p* = 0.043 and *p* = 0.010).

For our third aim, Table 4 reports Pearson’s correlations between the main variables assessed in the present study.

As can be noted, intention to vaccinate was positively and significantly correlated with perceived risk, pro-sociality, fear of COVID-19, use of preventive behaviors, trust in government, trust in science, and trust in medical professionals. Thus, acceptance of COVID-19 vaccines was higher in those participants who perceived more risk, were more prosocial, had more fear of the virus, used preventive behaviors more frequently, and were more trustful of government, scientists, and medical practitioners. In addition, intention to vaccinate was negatively and significantly associated with belief in misinformation; thus, participants who had higher levels of belief in COVID-19-related misinformation stated that they were less likely to vaccinate (as compared to participants who had lower levels of belief in misinformation). To determine the psychological predictors of the intention to vaccinate, we ran a hierarchical regression analysis, using the simultaneous method (see Table 5).

Demographic factors were entered in the first step as a series of dummy variables, to control for their influence. The overall model was significant (*F*(26, 942) = 17.14, *p* < 0.001). Demographic variables explained 4.7% of the variance in the intention to vaccinate (*F*(16, 952) = 2.95, *p* < 0.001); specifically, vaccination rates increased for participants who were older than 50 years and had a postgraduate degree, but decreased for participants who were females, married, or divorced/widowed. Psychological variables explained 27.4% of the variance (*F*(10, 942) = 38.02, *p* < 0.001): vaccination rates increased for participants who had fear of COVID-19, used more preventive measures, and were trustful of science, government, and medical professionals, whereas it decreased for participants who had high levels of belief in misinformation.

## 4. Discussion

In the present study, we investigated the intention to get vaccinated against COVID-19 in Italy in the period between March and May 2021. With respect to this first aim, we found that vaccine acceptance rates were substantially higher than those previously reported by Palamenghi et al. [4]. The overall percentage of participants who reported to be “very likely” and “absolutely likely” to vaccinate was 86.9% (*N* = 850). Likewise, the percentage of participants who were “very likely” and “absolutely likely” to recommend vaccination for their family members was 89.0% (*N* = 870). Similar estimates have been recently reported by Kerr et al. [6], who found that 85% of Italian respondents were likely to be vaccinated and 88% recommended vaccination to vulnerable friends or family members, and by Barello et al. [29], who estimated that 86% of Italian university students would choose to have a vaccination against COVID-19. This rapid increase in acceptance rates was expected, since the growing availability of COVID-19 vaccines has been accompanied by a widespread campaign of public health messaging specifically tailored to address people’s negative attitudes towards vaccines. Our data are also consistent with the high number of doses administered so far in Italy: as reported in the Introduction Section, about 85% of the population over 12 years of age has been immunized with two doses and about 89% has received at least one dose. On the other hand, the relatively low rates of participants who declared to be hesitant about vaccination could be the result of the period in which our data were collected (in Italy, the overall number of infections was rapidly decreasing during the spring and fall of 2021 and this trend was primarily attributed by the authorities to the benefits of vaccination) and the characteristics of the sample that was recruited for the present study (most of our participants were young individuals aged between 18 and 30, with high educational levels, who might be particularly willing to get vaccination).

Our second aim was to determine the influence of individual differences in demographic variables on the intention to be vaccinated. We found that males and females did not differ in a direct comparison; however, in the following regression analysis, being female was associated with a reduced intention to get vaccinated against COVID-19. This is a common result which has been further confirmed by a recent meta-analysis [2] and may be attributed to the fact that females have typically high levels of mistrust about vaccine benefits and more negative concerns about future unforeseen side effects, which in turn are important determinants of the willingness to be vaccinated [14]. In the present study, this tendency might have been exacerbated by the fact that females are more likely to use social media and were therefore overrepresented in our sample. Replicating the conclusions reached by Palamenghi et al. [4], we found that the middle-age group (41–50 years) was less likely to vaccinate, as compared to both the younger (18–30 years) and older (>61 years) groups. On the one hand, this outcome confirms the idea that elder people are aware of being more susceptible to the negative consequences of COVID-19 [30] and therefore more willing to vaccinate [31]. On the other hand, the present results echo previous data showing that Italian parents older than 35 years of age exhibited more hesitancy about the vaccination of their children and were less compliant with vaccination recommendations compared to parents younger than 35 years [32]. Surprisingly, statistical analyses revealed that the intention to be vaccinated was significantly higher in participants who were single than in those who were married or divorced/widowed. Furthermore, vaccine acceptance rates were not significantly higher in participants who lived with others (family members, partners, friends, or housemates) than in those who lived alone. This is apparently in contrast with available evidence indicating that, in both the United Kingdom and the United States, more respondents would accept a vaccine to protect family, friends, or at-risk groups than to protect themselves [13]. A potential explanation may be that, in the present study, marital status was confounded with age, such that participants who were single came predominantly from the youngest group (18–30 years, 88%), i.e., a group who, as stated below, exhibited high levels of willingness to be vaccinated; in contrast, participants who were married or divorced/widowed came predominantly from the 41–50 year (25.9% and 30.8%, respectively) and 51–60 year age groups (28.7% and 41.0%, respectively), i.e., two groups in which intention to get vaccinated was substantially below the mean level of the whole sample (see Table 3).

With respect to the third aim, determining the roles of several psychological variables in predicting the intention to be vaccinated against COVID-19, our results are largely consistent with previously published findings. More specifically, we found that trust in government, trust in science, and trust in medical professionals were among the strongest psychological predictors of the intention to be vaccinated. In agreement, people with high levels of trust in science have been shown to be more compliant with COVID-19 prevention guidelines [33,34,35,36,37] and more likely to get vaccinated against COVID-19 [4,6,38]. In the cross-national study by Kerr et al. [6], trust in medical doctors and nurses predicted vaccine acceptance in Italy, together with perceived infection risk and worry about the virus. Similarly, willingness to be vaccinated correlated with trust in scientific research in the study by Palamenghi et al. [4].

Interestingly, we found that fear of COVID-19, but not perceived risk, was associated with increased vaccine uptake in the regression analysis, suggesting that the affective component of risk perception was more important than the cognitive component in predicting participants’ behaviors during the pandemic [12]. Previous research examining the role of these two factors reported mixed findings. Studies conducted during the first wave typically found significant effects of perceived severity of COVID-19 on vaccination intent [10,32]. Gagneux-Brunon et al. [9], for example, showed that fear of COVID-19 and individual perceived risk were both positively correlated with vaccine acceptance in a sample of French healthcare workers (also see Detoc et al. [39]). On the other hand, Qiao, Tam, and Li [40] found that fear of COVID-19, but not perceived susceptibility to the infection, was associated with increased willingness to be vaccinated in a sample of college students in North Carolina (also see [41]). It seems likely that variables such as the period in which the surveys were conducted and the demographic characteristics of the recruited samples might explain these discrepancies. Specifically, our study was performed during the second wave of the COVID-19 infection and most of our respondents were young people, aged between 18 and 30. These two factors might have resulted in relatively low levels of perceived risk, which in turn contributed to the non-significant association with intention to get vaccinated.

While the effects of trust variables were positive, a variable which reduced participants’ intention to be vaccinated in our study was susceptibility to misinformation. This is in line with the conclusions obtained by a randomized controlled trial conducted by Loomba et al. [13], who found that recent exposure to misinformation induced a decline in vaccination intent of 6.2 percentage points in the UK and 6.4 percentage points in the USA. Similar findings have been reported by Roozenbeek et al. [15], who showed that, across five different countries (UK, Ireland, USA, Spain, and Mexico), increased susceptibility to misinformation led to a significant decrease in people’s willingness to get vaccinated against the virus and to recommend the vaccine to vulnerable friends and family.

Our findings have practical implications for developing interventions aimed at increasing the acceptance of COVID-19 vaccines. First, since trust in science and trust in medical professionals play a key role in predicting participants’ willingness to be vaccinated, public health institutions should try to increase the feeling of cooperation between scientists and citizens [4]. The scientific community should create a dialogue aimed at educating and sensitizing common people towards the logic and the limits of scientific research [42]. The mission of all scientists involved in the battle against COVID-19 is not simply to explain the reasons that justify the adoption of restraining measures, but to help create an enduring debate in which public concerns about the safety and effectiveness of vaccines can be expressed and properly addressed. In Italy, where most of the adult population has already received two doses, the establishment of such a climate would be particularly helpful in increasing acceptance of the so-called booster dose—which is still low (66%), according to a recent poll [43]. Along the same lines, a successful COVID-19 vaccination campaign must actively fight against the spreading of misinformation, which seems to be especially fast on social media [44]. This issue is particularly important since previous studies have shown that even brief exposures to misinformation can result in long-lasting negative effects on intention to get vaccinated [45]. Social media such as Facebook and Twitter have already adopted algorithms and fact-checking platforms to ensure amplification of right and trustable sources, to direct users to reliable websites and to filter out fake news about COVID-19 [46]. In addition, experimental evidence suggests that people tend to endorse false claims about COVID-19 because they do not spend sufficient time evaluating content accuracy and that a simple reminder at the beginning of presentation is sufficient to boost the level of trust discernment in participants who subsequently share information on social media [47].

The present study has both strengths and limitations. The strengths are that we provided an updated picture of the vaccination intentions at the beginning of 2021, in a period in which the Italian government had just launched the immunization campaign, whereas previous studies were mostly conducted during the first lockdown phase between March and May 2020 [4]. Moreover, in line with previous international research [12], we assessed a wide array of predictors, covering the cognitive, emotional, experiential, and sociocultural implications of the current pandemic, whereas previous studies have been often focused on single aspects. Lastly, it is interesting that participants were recruited mainly using social media, considering that, as previously noted, the spreading of misinformation seems to be especially fast on these media. Regarding limitations, our sample was not representative of the general Italian population because participants were recruited through different social media and were therefore mostly young, between 18 and 30 years of age, with high education levels. Second, the method was cross-sectional and correlational in nature, which means that we could not determine whether demographic and psychological factors were causally related to intention to be vaccinated. Lastly, despite the large number of predictors included in our survey, not all relevant variables were considered, such as political ideology [48], personality traits [49,50], and general vaccine attitudes and beliefs [9,10]. Future studies should consider these variables to better understand the multifaceted process underlying people’s intention to get vaccinated.

## Figures and Tables

**Table 1:** Demographic properties of the sample recruited for the present study, as compared to the Italian population.

	Our Sample	Italian Population ^a^
Gender		
Females	558 (57.6%)	51.3%
Males	411 (42.4%)	49.7%
Age		
18–30 years	641 (66.0%)	14.9%
31–40 years	91 (9.4%)	11.3%
41–50 years	97 (10.0%)	14.7%
51–60 years	94 (9.7%)	15.9%
>61 years	48 (4.9%)	30.2%
Education		
High school or less	465 (47.8%)	85.1%
Bachelor’s degree	157 (16.2%)	3.8%
Master’s degree	223 (23.0%)	10.7%
Postgraduate	126 (13.0%)	0.4%
Marital status		
Single	681 (70.1%)	42.9%
Married	251 (25.8%)	46.6%
Divorced/widowed	39 (4.0%)	10.5%
Living condition		
Alone	109 (11.2%)	32.9%
Family/Partner	795 (81.9%)	63.2%
Friends/Housemates	67 (6.9%)	3.9%
Region		
Central Italy	713 (73.4%)	19.9%
North Italy	148 (15.2%)	46.4%
South Italy	110 (11.3%)	33.7%
Type of area		
White/Yellow	350 (36.0%)	-
Orange	128 (13.2%)	-
Red	493 (50.8%)	-

Note ^a^: Data taken from https://www.istat.it/it/censimenti (accessed on 29 December 2021).

**Table 2:** Descriptive statistics for the variables measured in the present study.

Measures	M	SD	Min	Max
Intention to vaccinate	9.13	1.79	2.00	10.00
Perceived risk	12.00	2.87	3.00	19.00
Pro-sociality	5.77	1.45	1.00	7.00
Fear of COVID-19	14.94	5.52	7.00	35.00
Direct experience	0.48	0.49	0.00	1.00
Use of preventive behaviors	38.23	8.03	10.00	50.00
Misinformation (number)	2.52	1.49	1.00	10.00
Misinformation (belief)	1.26	0.50	1.00	4.00
Trust in government	3.32	1.54	1.00	7.00
Trust in science	4.17	0.90	1.00	5.00
Trust in medical professionals	4.34	0.78	1.00	5.00

**Table 3:** Means (and standard deviations) for intention to vaccinate (z-scores), as a function of gender, age, education, marital status, living condition, region, and type of area, together with the results of statistical analyses (*t*-test or F-test).

Categories	Intention to Vaccinate (z-Scores)	*t*-Test/F-Test
Gender		
Females (*N* = 558)	0.04 (0.97)	1.57
Males (*N* = 411)	−0.05 (1.01)	
Age		
18–30 years (*N* = 641)	0.05 (0.89)	3.77 **
31–40 years (*N* = 91)	−0.14 (1.12)	
41–50 years (*N* = 97)	−0.26 (1.32)	
51–60 years (*N* = 94)	−0.11 (1.21)	
>61 years (*N* = 48)	0.23 (0.81)	
Education		
High school or less (*N* = 465)	−0.01 (1.03)	0.27
Bachelor’s degree (*N* = 157)	−0.03 (1.01)	
Master’s degree (*N* = 223)	0.01 (0.95)	
Postgraduate (*N* = 126)	0.06 (0.94)	
Marital status		
Single (*N* = 681)	0.08 (0.87)	9.43 ***
Married (*N* = 251)	−0.15 (1.17)	
Divorced/widowed (*N* = 39)	−0.44 (1.56)	
Living condition		
Alone (*N* = 109)	0.07 (0.97)	1.55
Family/Partner (*N* = 795)	−0.02 (1.01)	
Friends/Housemates (*N* = 67)	0.17 (0.84)	
Region		
Central Italy (*N* = 713)	0.02 (0.97)	1.03
North Italy (*N* = 148)	−0.10 (1.15)	
South Italy (*N* = 110)	0.01 (0.92)	
Type of area		
White/Yellow (*N* = 350)	0.01 (0.94)	4.14 **
Orange (*N* = 128)	−0.23 (1.21)	
Red (*N* = 493)	0.05 (0.97)	

Note. **: *p* ≤ 0.01; ***: *p* ≤ 0.001.

**Table 4:** Pearson’s correlations between all variables (*N* = 978).

Total Sample	1	2	3	4	5	6	7	8	9	10	11
1. Intention to vaccinate	1.00										
2. Perceived risk	0.15 **	1.00									
3. Pro-sociality	0.23 **	0.16 **	1.00								
4. Fear of COVID-19	0.12 **	0.36 **	0.08 **	1.00							
5. Direct experience	0.02	0.18 **	0.01	0.07 *	1.00						
6. Use of preventive behaviors	0.20 **	0.12 **	0.20 **	0.16 **	−0.01	1.00					
7. Misinformation (number)	0.05	0.09 **	0.04	0.04	0.03	0.07 *	1.00				
8. Misinformation (belief)	−0.22 **	−0.06 †	−0.10 **	0.03	−0.04	−0.14 **	−0.05	1.00			
9. Trust in government	0.29 **	0.05	0.25 **	0.05	−0.05	0.07 *	0.01	−0.04	1.00		
10. Trust in science	0.47 **	0.14 **	0.32 **	−0.01	0.02	0.23 **	0.09 *	−0.14 **	0.36 **	1.00	
11. Trust in medical professionals	0.39 **	0.13 **	0.28 **	0.03	−0.02	0.22 **	0.08 *	−0.16 **	0.28 **	0.61 **	1.00

Note. *: *p* ≤ 0.05; **: *p* ≤ 0.01; †: 0.06 < *p* < 0.10.

**Table 5:** Simultaneous regression predicting the intention to vaccinate.

Steps	Predictors	β	*t*
Step 1	Gender		
	Males	- ^a^	-
	Females	−0.08	−2.56 **
	Age		
	18–30 years	- ^a^	-
	31–40 years	0.02	0.59
	41–50 years	0.04	1.27
	51–60 years	0.09	2.41 *
	>61 years	0.17	4.99 **
	Education		
	High school or less	- ^a^	-
	Bachelor’s degree	−0.00	−0.12
	Master’s degree	0.01	0.54
	Postgraduate	0.06	2.05 *
	Marital status		
	Single	- ^a^	-
	Married	−0.14	−3.23 ***
	Divorced/widowed	−0.09	−2.60 **
	Living condition		
	Alone	- ^a^	-
	Family/Partner	0.01	0.24
	Friends/Housemates	0.01	0.27
	Region		
	Central Italy	- ^a^	-
	North Italy	0.00	0.02
	South Italy	−0.02	−0.78
	Area		
	Red	- ^a^	-
	Orange	−0.02	−0.58
	White/Yellow	−0.04	−1.17
Step 2	Psychological predictors		
	Perceived risk	0.05	1.64
	Pro-sociality	0.02	0.73
	Fear of COVID-19	0.11	3.73 ***
	Direct experience	−0.00	−0.04
	Use of preventive behaviors	0.06	2.08 *
	Misinformation (number)	−0.00	−0.09
	Misinformation (belief)	−0.16	−5.55 ***
	Trust in government	0.11	3.86 ***
	Trust in Science	0.29	7.91 ***
	Trust in medical professionals	0.14	4.07 ***

Note. *: *p* ≤ 0.05; **: *p* ≤ 0.01; ***: *p* ≤ 0.001; ^a^: reference category.

## Data Availability

The data presented in this study are available upon request from the corresponding author.

## References

[1] Ipsos. https://www.ipsos.com/en/covid-19-vaccination-intent-has-soared-across-world.

[2] Robinson E., Jones A., Lesser I., Daly M. (2021). International estimates of intended uptake and refusal of COVID-19 vaccines: A rapid systematic review and meta-analysis of large nationally representative samples. Vaccine.

[3] (2021). Our World in Data. https://ourworldindata.org/covid-vaccinations.

[4] Palamenghi L., Barello S., Boccia S., Graffigna G. (2020). Mistrust in biomedical research and vaccine hesitancy: The forefront challenge in the battle against COVID-19 in Italy. Eur. J. Epidemiol..

[5] Caserotti M., Girardi P., Rubaltelli E., Tasso A., Lotto L., Gavaruzzi T. (2021). Associations of COVID-19 risk perception with vaccine hesitancy over time for Italian residents. Soc. Sci. Med..

[6] Kerr J.R., Schneider C.R., Recchia G., Dryhurst S., Sahlin U., Dufouil C., Arwidson P., Freeman A.L., van der Linden S. (2021). Correlates of intended COVID-19 vaccine acceptance across time and countries: Results from a series of cross-sectional surveys. BMJ Open.

[7] Pivetti M., Di Battista S., Paleari F.G., Hakoköngäs E. (2021). Conspiracy beliefs and attitudes toward COVID-19 vaccinations. J. Pac. Rim Psychol..

[8] Cadeddu C., Sapienza M., Castagna C., Regazzi L., Paladini A., Ricciardi W., Rosano A. (2021). Vaccine Hesitancy and Trust in the Scientific Community in Italy: Comparative Analysis from Two Recent Surveys. Vaccines.

[9] Gagneux-Brunon A., Detoc M., Bruel S., Tardy B., Rozaire O., Frappe P., Botelho-Nevers E. (2021). Intention to get vaccinations against COVID-19 in French healthcare workers during the first pandemic wave: A cross-sectional survey. J. Hosp. Infect..

[10] Sherman S.M., Smith L.E., Sim J., Amlôt R., Cutts M., Dasch H., Rubin G.J., Sevdalis N. (2020). COVID-19 vaccination intention in the UK: Results from the COVID-19 vaccination acceptability study (CoVAccS), a nationally representative cross-sectional survey. Hum. Vaccines Immunother..

[11] Van der Linden S. (2015). The social-psychological determinants of climate change risk perceptions: Towards a comprehensive model. J. Environ. Psychol..

[12] Dryhurst S., Schneider C.R., Kerr J., Freeman A.L.J., Recchia G., van der Bles A.M., Spiegelhalter D., van der Linden S. (2020). Risk perceptions of COVID-19 around the world. J. Risk Res..

[13] Loomba S., de Figueiredo A., Piatek S.J., de Graaf K., Larson H.J. (2021). Measuring the impact of COVID-19 vaccine misinformation on vaccination intent in the UK and USA. Nat. Hum. Behav..

[14] Paul E., Steptoe A., Fancourt D. (2021). Attitudes towards vaccines and intention to vaccinate against COVID-19: Implications for public health communications. Lancet Reg. Health Eur..

[15] Roozenbeek J., Schneider C.R., Dryhurst S., Kerr J., Freeman A.L.J., Recchia G., van der Bles A.M., van der Linden S. (2020). Susceptibility to misinformation about COVID-19 around the world. R. Soc. Open Sci..

[16] Lazarus J.V., Wyka K., Rauh L., Rabin K., Ratzan S., Gostin L.O., Larson H.J., El-Mohandes A. (2020). Hesitant or Not? The Association of Age, Gender, and Education with Potential Acceptance of a COVID-19 Vaccine: A Country-level Analysis. J. Health Commun..

[17] Frank K., Arim R. Statistics Canada. https://www150.statcan.gc.ca/n1/pub/45-28-0001/2020001/article/00043-eng.htm.

[18] Reiter P.L., Pennell M.L., Katz M.L. (2020). Acceptability of a COVID-19 vaccine among adults in the United States: How many people would get vaccinated?. Vaccine.

[19] Wong L.P., Alias H., Wong P.-F., Lee H.Y., Abubakar S. (2020). The use of the health belief model to assess predictors of intent to receive the COVID-19 vaccine and willingness to pay. Hum. Vaccines Immunother..

[20] Rosenstock I.M., Strecher V.J., Becker M.H. (1988). Social Learning Theory and the Health Belief Model. Health Educ. Q..

[21] Floyd D.L., Prentice-Dunn S., Rogers R.W. (2000). A Meta-Analysis of Research on Protection Motivation Theory. J. Appl. Soc. Psychol..

[22] Yahaghi R., Ahmadizade S., Fotuhi R., Taherkhani E., Ranjbaran M., Buchali Z., Jafari R., Zamani N., Shahbazkhania A., Simiari H. (2021). Fear of COVID-19 and Perceived COVID-19 Infectability Supplement Theory of Planned Behavior to Explain Iranians’ Intention to Get COVID-19 Vaccinated. Vaccines.

[23] Jung H., Albarracín D. (2020). Concerns for others increases the likelihood of vaccination against influenza and COVID-19 more in sparsely rather than densely populated areas. Proc. Natl. Acad. Sci. USA.

[24] Yu Y., Luo S., Mo P.K.-H., Wang S., Zhao J., Zhang G., Li L., Li L., Lau J.T.-F. (2021). Prosociality and Social Responsibility Were Associated With Intention of COVID-19 Vaccination Among University Students in China. Int. J. Health Policy Manag..

[25] Rosman T., Adler K., Barbian L., Blume V., Burczeck B., Cordes V., Derman D., Dertli S., Glas H., Heinen V. (2021). Protect ya Grandma! The Effects of Students’ Epistemic Beliefs and Prosocial Values on COVID-19 Vaccination Intentions. Front. Psychol..

[26] Ahorsu D.K., Lin C.-Y., Imani V., Saffari M., Griffiths M.D., Pakpour A.H. (2020). The Fear of COVID-19 Scale: Development and Initial Validation. Int. J. Ment. Health Addict..

[27] Soraci P., Ferrari A., Abbiati F.A., Del Fante E., De Pace R., Urso A., Griffiths M.D. (2020). Validation and Psychometric Evaluation of the Italian Version of the Fear of COVID-19 Scale. Int. J. Ment. Health Addict..

[28] Lee J.J., Kang K.-A., Wang M.P., Zhao S.Z., Wong J.Y.H., O’Connor S., Yang S.C., Shin S. (2020). Associations Between COVID-19 Misinformation Exposure and Belief With COVID-19 Knowledge and Preventive Behaviors: Cross-Sectional Online Study. J. Med. Internet Res..

[29] Barello S., Nania T., Dellafiore F., Graffigna G., Caruso R. (2020). ‘Vaccine hesitancy’ among university students in Italy during the COVID-19 pandemic. Eur. J. Epidemiol..

[30] Lazarus J.V., Ratzan S.C., Palayew A., Gostin L.O., Larson H.J., Rabin K., Kimball S., El-Mohandes A. (2021). A global survey of potential acceptance of a COVID-19 vaccine. Nat. Med..

[31] Ruiz J.B., Bell R.A. (2021). Predictors of intention to vaccinate against COVID-19: Results of a nationwide survey. Vaccine.

[32] Giambi C., Fabiani M., D’Ancona F., Ferrara L., Fiacchini D., Gallo T., Martinelli D., Pascucci M.G., Prato R., Filia A. (2018). Parental vaccine hesitancy in Italy—Results from a national survey. Vaccine.

[33] Bicchieri C., Fatas E., Aldama A., Casas A., Deshpande I., Lauro M., Parilli C., Spohn M., Pereira P., Wen R. (2021). In science we (should) trust: Expectations and compliance across nine countries during the COVID-19 pandemic. PLoS ONE.

[34] Dohle S., Wingen T., Schreiber M. (2020). Acceptance and adoption of protective measures during the COVID-19 pandemic: The role of trust in politics and trust in science. Soc. Psychol. Bull..

[35] Hromatko I., Tonković M., Vranic A. (2021). Trust in Science, Perceived Vulnerability to Disease, and Adherence to Pharmacological and Non-pharmacological COVID-19 Recommendations. Front. Psychol..

[36] Pagliaro S., Sacchi S., Pacilli M.G., Brambilla M., Lionetti F., Bettache K., Bianchi M., Biella M., Bonnot V., Boza M. (2021). Trust predicts COVID-19 prescribed and discretionary behavioral intentions in 23 countries. PLoS ONE.

[37] Plohl N., Musil B. (2020). Modeling compliance with COVID-19 prevention guidelines: The critical role of trust in science. Psychol. Health Med..

[38] Viswanath K., Bekalu M., Dhawan D., Pinnamaneni R., Lang J., McLoud R. (2021). Individual and social determinants of COVID-19 vaccine uptake. BMC Public Health.

[39] Detoc M., Bruel S., Frappe P., Tardy B., Botelho-Nevers E., Gagneux-Brunon A. (2020). Intention to participate in a COVID-19 vaccine clinical trial and to get vaccinated against COVID-19 in France during the pandemic. Vaccine.

[40] Qiao S., Tam C.C., Li X. (2021). Risk Exposures, Risk Perceptions, Negative Attitudes toward General Vaccination, and COVID-19 Vaccine Acceptance among College Students in south Carolina. Am. J. Health Promot..

[41] Chu H., Liu S. (2021). Integrating health behavior theories to predict American’s intention to receive a COVID-19 vaccine. Patient Educ. Couns..

[42] Provenzi L., Barello S. (2020). The Science of the Future: Establishing a Citizen-Scientist Collaborative Agenda After Covid-19. Front. Public Health.

[43] Ipsos. https://www.ipsos.com/it-it/covid-terza-dose-vaccino-maggioranza-intervistati-riceverebbe-dose-richiamo.

[44] Vosoughi S., Roy D., Aral S. (2018). The spread of true and false news online. Science.

[45] Pluviano S., Watt C., Della Sala S. (2017). Misinformation lingers in memory: Failure of three pro-vaccination strategies. PLoS ONE.

[46] Brindha M.D., Jayaseelan R., Kadeswara S. (2020). Social Media Reigned by Information or Misinformation About COVID-19: A Phenomenological Study. Alochana Chakra J..

[47] Pennycook G., McPhetres J., Zhang Y., Lu J.G., Rand D.G. (2020). Fighting COVID-19 Misinformation on Social Media: Experimental Evidence for a Scalable Accuracy-Nudge Intervention. Psychol. Sci..

[48] Calvillo D.P., Ross B.J., Garcia R.J.B., Smelter T.J., Rutchick A.M. (2020). Political Ideology Predicts Perceptions of the Threat of COVID-19 (and Susceptibility to Fake News About It). Soc. Psychol. Pers. Sci..

[49] Hampshire A., Hellyer P.J., Soreq E., Mehta M.A., Ioannidis K., Trender W., Grant J.E., Chamberlain S.R. (2021). Associations between dimensions of behaviour, personality traits, and mental-health during the COVID-19 pandemic in the United Kingdom. Nat. Commun..

[50] Zajenkowski M., Jonason P.K., Leniarska M., Kozakiewicz Z. (2020). Who complies with the restrictions to reduce the spread of COVID-19?: Personality and perceptions of the COVID-19 situation. Pers. Individ. Differ..

